# Interface-Engineered RuP_2_/Mn_2_P_2_O_7_ Heterojunction on N/P Co-Doped Carbon for High-Performance Alkaline Hydrogen Evolution

**DOI:** 10.3390/ma18133065

**Published:** 2025-06-27

**Authors:** Wenjie Wu, Wenxuan Guo, Zeyang Liu, Chenxi Zhang, Aobing Li, Caihua Su, Chunxia Wang

**Affiliations:** 1 State Key Laboratory of Heavy Oil Processing, College of New Energy and Materials, China University of Petroleum (Beijing), Beijing 102249, China; wuwenjie21@mails.ucas.ac.cn (W.W.); gwx15630163202@163.com (W.G.); liuzy185z@163.com (Z.L.); tevth_12138@163.com (C.Z.); 18240398133@163.com (A.L.); 2 Beijing Future Hydrogen Technology Co., Ltd., Beijing 102209, China; chsu@future-h2.com

**Keywords:** transition metal phosphides, heterostructure, electrocatalytic hydrogen evolution, three-dimensional nitrogen-doped porous carbon

## Abstract

Developing efficient and durable electrocatalysts for the alkaline hydrogen evolution reaction (HER) is crucial for sustainable hydrogen production. Herein, we report a novel RuP_2_/Mn_2_P_2_O_7_ heterojunction anchored on a three-dimensional nitrogen and phosphorus co-doped porous carbon (RuP_2_/Mn_2_P_2_O_7_/NPC) framework as a high-performance HER catalyst, synthesized via a controlled pyrolysis–phosphidation strategy. The heterostructure achieves uniform dispersion of ultrafine RuP_2_/Mn_2_P_2_O_7_ heterojunctions with well-defined interfaces. Furthermore, phosphorus doping restructures the electronic configuration of Mn and Ru species at the RuP_2_/Mn_2_P_2_O_7_ heterointerface, enabling enhanced catalytic activity through the accelerated electron transfer and kinetics of the HER. This RuP_2_/Mn_2_P_2_O_7_/NPC catalyst exhibits exceptional HER activity with 1 M KOH, requiring only 69 mV of overpotential to deliver 10 mA·cm^−2^ and displaying a small Tafel slope of 69 mV·dec^−1^, rivaling commercial 20% Pt/C. Stability tests reveal negligible activity loss over 48 h, underscoring the robustness of the heterostructure. The RuP_2_/Mn_2_P_2_O_7_ heterojunction demonstrates markedly reduced overpotentials for the electrochemical HER process, highlighting its enhanced catalytic efficiency and improved cost-effectiveness compared to the conventional catalytic systems. This work establishes a strategy for designing a transition metal phosphide heterostructure through interfacial electronic modulation, offering broad implications for energy conversion technologies.

## 1. Introduction

Hydrogen energy has emerged as a global research focus in the ongoing energy transition, owing to its high density and clean combustion properties [1,2]. Among various hydrogen production technologies, water electrolysis has attracted considerable attention due to its efficiency and sustainability [3,4,5,6,7]. As a critical half-reaction in this process, the hydrogen evolution reaction (HER) plays a pivotal role, with the performance of HER catalysts directly influencing the overall efficiency and cost of hydrogen production [8,9,10,11]. Current studies have shown that platinum (Pt)-based catalysts exhibit outstanding HER activity in both acidic and alkaline electrolytes [12]. However, their high cost and limited natural reserves pose significant barriers to large-scale commercialization [13,14]. Consequently, the development of alternative catalysts based on transition metals with excellent catalytic performance has emerged as a critical area of research in the field of new energy materials [15,16].

Ruthenium (Ru)-based materials, with advantages such as high activity and low cost, are expected to become effective alternatives to Pt-based catalysts in alkaline HER [17,18,19]. However, in alkaline environments, Ru-based electrocatalysts frequently exhibit an electron-rich state, leading to strong adsorption of active hydrogen species (*H) and, consequently, hindering HER activity [20,21,22]. Transition metal phosphides (TMPs) have attracted considerable attention for the hydrogen evolution reaction (HER) under alkaline conditions, which can be attributed to their unique electronic structures and promising catalytic properties [23,24,25,26]. Recent studies have shown that the construction of metallic phosphide active sites can optimize the adsorption of reaction intermediates, thereby enhancing HER activity [27,28]. However, monometallic phosphide catalysts still suffer from several limitations, including a limited number of active sites, suboptimal electron transfer efficiency, and poor long-term stability [29]. To overcome these challenges, the construction of heterostructures has emerged as an effective strategy [30,31]. Through rationally integrating different metal phosphides, the complementary properties of each component can be exploited to achieve synergistic catalytic effects. Such heterostructures can increase both the number and diversity of active sites, while electronic interactions at the interface optimize charge distribution and facilitate charge transfer—thereby significantly enhancing overall catalytic performance [32]. Despite these advantages, ultrafine TMPs tend to agglomerate due to their high surface free energy [33]. Consequently, the controllable synthesis of stably dispersed TMPs nanoclusters remains a major challenge for efficient HER catalysis.

In this work, we fabricated a RuP_2_/Mn_2_P_2_O_7_ heterojunction supported on a three-dimensional nitrogen and phosphorus co-doped porous carbon (RuP_2_/Mn_2_P_2_O_7_/NPC) matrix. The three-dimensional-NPC not only provides excellent electrical conductivity and structural stability, but also offers abundant anchoring sites for the uniform dispersion of metal phosphides, thereby enhancing the utilization of active components. Electrochemical investigations confirmed that phosphorus doping and the formation of the RuP_2_/Mn_2_P_2_O_7_ heterojunction enhance HER performance via increasing the electrochemically active surface area (ECSA), requiring a low overpotential of 69 mV at 10 mA·cm^−2^ and exhibiting an ultra-low Tafel slope of 69 mV·dec^−1^ under alkaline conditions. Furthermore, precise control over the phosphorus precursor concentration enables the formation of the RuP_2_/Mn_2_P_2_O_7_ heterojunction, which facilitates optimized electron and proton transport pathways during the HER process, thereby significantly improving catalytic activity in alkaline media. Notably, the catalyst demonstrates HER performance comparable to that of commercial 20% Pt/C, along with excellent electrochemical stability. This work offers an effective synthetic strategy for the development of highly active and durable catalysts for alkaline HER applications.

## 2. Experimental Procedure

### 2.1. Chemicals

All chemical reagents were of analytical grade and used without further purification. Melamine (99%) was purchased from Energy Chemical (Nottinghamshire, UK). Ruthenium (III) chloride trihydrate (98%), manganese acetate (98%), and ammonium dihydrogen phosphate (AR) were purchased from Aladdin Chemical Co., Ltd. (Shanghai, China). Manganese acetate was purchased from Innochem (Pyeongtaek, Republic of Korea). The 5% Nafion-521 dispersion was purchased from Alfa Aesar (Waltham, MA, USA). Potassium hydroxide was obtained from Sinopharm Chemical Reagent Co., Ltd. (Shanghai, China). Nafion-117 membrane was purchased from DuPont (Wilmington, DE, USA). High-purity Ar gas (99.99%) was purchased from Beijing Millennium Capital Gas Co., Ltd. (Beijing, China).

### 2.2. Synthesis of RuP_2_/Mn_2_P_2_O_7_/NPC and Control Catalysts

The synthesis of catalysts was carried out through the direct pyrolysis of pre-organized precursors. For RuP_2_/Mn_2_P_2_O_7_/NPC, melamine foam (3 cm × 5 cm× 5 cm) was ultrasonically cleaned in ethanol and deionized water for 30 min. After drying for 12 h at 80 °C, the melamine foam substrate was immersed in an aqueous solution containing melamine powder, ruthenium trichloride trihydrate, and manganese acetate in a molar ratio of 1.1:1:1, followed by the addition of 100 mM ammonium dihydrogen phosphate. After stirring for 24 h, the impregnated melamine foam was dried at 80 °C until complete solvent evaporation was achieved. The obtained melamine foam was carbonized by heating at 500 °C for 2 h and then at 800 °C for 2 h with a heating rate of 3 °C/min under N_2_ atmosphere. The product was etched with 1 M HCl solution for 12 h at room temperature and dried to obtain the final RuP_2_/Mn_2_P_2_O_7_/NPC catalyst.

RuMn@NP_50_C and RuMn@NP_150_C were synthesized following the same procedure, except that the concentrations of ammonium dihydrogen phosphate were 50 and 150 mM, respectively. The synthesis of RuMn@NC followed the same procedure as that of RuP_2_/Mn_2_P_2_O_7_/NPC, except that no ammonium dihydrogen phosphate was added during the precursor preparation step. All other reagents, processing conditions, and pyrolysis steps remained unchanged. In addition, the single-metal catalysts (Mn@NP_100_C and Ru@NP_100_C) were prepared following the same procedure, except that only the corresponding metal precursor was added. The metal-free NP_100_C catalyst was synthesized following an identical procedure but without the addition of a metal precursor or ammonium dihydrogen phosphate solution.

### 2.3. Characterization

The scanning electron microscopy (SEM) and transmission electron microscopy (TEM) images were obtained via Hitachi S-4800 (JEOL, Tokyo, Japan) (at 30 kV) and JEM-2100F (JEOL, Tokyo, Japan) (at 300 kV), respectively. Powder X-ray diffraction (PXRD) patterns were collected on a RigakuD/max 2500 Pc using Cu Kα radiation (λ = 1.5406 Å) generated by a Cu X-ray tube operating at 40 kV and 40 mA. X-ray photoelectron spectroscopy (XPS) measurements were performed on a VG-ESCALAB250XI system (Thermo Scientific, Waltham, MA, USA). The particle sizes were measured and statistically analyzed using Nano Measurer 1.2.5 software based on the high-resolution TEM images.

### 2.4. Electrocatalytic Measurement

The electrochemical hydrogen evolution reaction performance of RuP_2_/Mn_2_P_2_O_7_/NPC was evaluated in a customized H-type cell under ambient conditions using a three-electrode configuration. The system consisted of a carbon fiber paper working electrode, a platinum foil counter electrode (1 cm × 1 cm), and a saturated Hg/Hg_2_Cl_2_ (SCE) reference electrode. Electrochemical measurements were recorded using a CHI730e workstation (Shanghai Chenhua Instrument Co., Ltd., Shanghai, China). The catalyst dispersion was prepared by dispersing 2 mg of catalyst powder in a mixture of 220 µL isopropyl alcohol, 720 µL deionized water, and 60 µL Nafion-521 solution, followed by sonication for 1 h. The resulting catalyst ink was drop-cast onto either a glassy carbon electrode or a carbon paper electrode (1 × 1 cm^2^). The electrolyte solutions were tested in 1 M KOH. Each compartment of the H-type cell contained 45 mL of electrolyte, which was purged with high-purity Argon for 15 min before measurements. Linear sweep voltammetry (LSV) was performed at a scan rate of 10 mV/s, which was employed to directly determine the overpotential (η) and the reaction kinetics of the HER. The electrochemical active surface area (ECSA), a critical indicator of the density of active sites, was calculated using the following equation:

(1) $$ECSA=\frac{C_{dl}}{C_{sp}}$$

where the C_dl_ is the double-layer capacitance, and C_sp_ is the specific capacitance per unit area-typically ranging from 40 μF·cm^−2^.

## 3. Results and Discussion

### 3.1. Synthesis and Characterization of RuP_2_/Mn_2_P_2_O_7_/NPC Catalyst

**Scheme 1:**
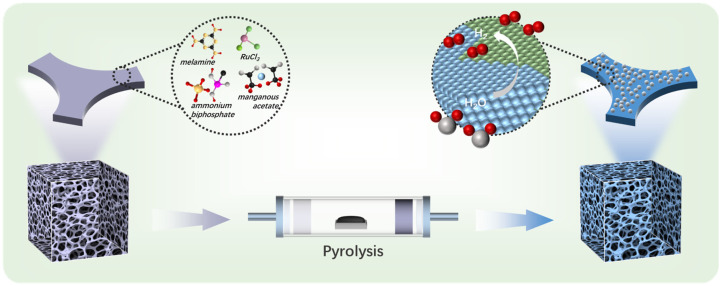
Schematic illustration of the preparation of RuP_2_/Mn_2_P_2_O_7_/NPC via a high-temperature pyrolysis–phosphidation strategy.

The RuP_2_/Mn_2_P_2_O_7_ heterojunction catalyst was synthesized through a high-temperature pyrolysis–phosphidation strategy using a melamine foam precursor pre-adsorbed with ruthenium and manganese ions. The precursor mixture consisted of melamine powder, ruthenium trichloride trihydrate, manganese acetate, ammonium dihydrogen phosphate, and melamine foam. The melamine powder served as the polymerization precursor, metal salts provided metal ions, the ammonium dihydrogen phosphate acted as a phosphorus source, and the melamine foam (MF) functioned as a template for creating hierarchical porous structures. Melamine foam, a lightweight porous material synthesized from melamine resin with a three-dimensional interconnected macroporous architecture and micrometer-scale surface asperities, demonstrates promising potential as a catalyst carrier capable of adsorbing significant quantities of metal precursors, making it promising for the synthesis of hierarchical porous structures. As illustrated in Scheme 1, the process began with melamine foam adsorbing metal ions, ammonium dihydrogen phosphate, and melamine at room temperature, forming Ru_x_Mn_y_(PO_4_)_Z_/MF precursors. As the pyrolysis temperature increases, Ru_x_Mn_y_(PO_4_)_Z_/MF precursors undergo further carbonization, ultimately forming a highly graphitized nitrogen-doped carbon framework loaded with a RuP_2_/Mn_2_P_2_O_7_ heterojunction under 800 °C. The final RuP_2_/Mn_2_P_2_O_7_/NPC catalyst with a hierarchical porous structure was obtained after HCl etching to remove the metal salts. The control catalysts (RuMn@NP_50_C, RuMn@NP_150_C, Mn@NP_100_C, Ru@NP_100_C, and NP_100_C) were synthesized following the same procedure with different precursors. By systematically varying the concentration of ammonium dihydrogen phosphate during synthesis (50, 100, and 150 mM), we found that only at 100 mM does the RuP_2_/Mn_2_P_2_O_7_ heterojunction form successfully. Lower concentrations lead to the incomplete transformation of Ru and Mn species, while higher concentrations promote the formation of amorphous or disordered phosphate phases that disrupt the crystalline heterostructure. This behavior likely arises from the balance of precursor stoichiometry, pyrolysis kinetics, and spatial confinement within the N,P-doped carbon matrix, which collectively influence phase selectivity and crystallinity [34,35,36,37].

**Figure 1:**
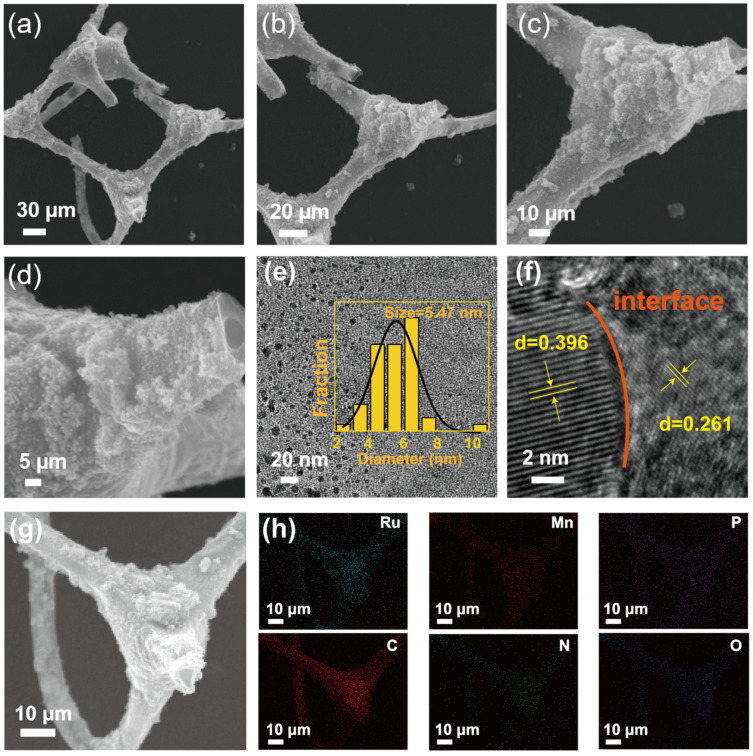
(**a**–**d**) Scanning electron microscope (SEM) images of RuP_2_/Mn_2_P_2_O_7_/NPC at different magnification. (**e**) High-resolution transmission electron microscopy (HRTEM) image of RuP_2_/Mn_2_P_2_O_7_/NPC (insert graph showing metal particle size). (**f**) Lattice spacing of metallic elements. (**g**) SEM and (**h**) energy-dispersive spectroscopy (EDS) elemental mapping images of RuP_2_/Mn_2_P_2_O_7_/NPC (Ru: cyan, Mn: pink, P: purple, C: red, N: green, O: blue).

The SEM images reveal that RuP_2_/Mn_2_P_2_O_7_/NPC possesses a three-dimensional nitrogen-doped porous carbon framework. As shown in Figure 1a–d, RuP_2_/Mn_2_P_2_O_7_ was uniformly dispersed on the porous carbon framework, and the MF maintained the three-dimensional porous structure after high-temperature heat treatment. The highly porous architecture facilitates efficient mass transport, promotes rapid electrolyte penetration, and enables effective gas bubble dissipation during HER processes. To facilitate an in-depth examination of the morphological properties, high-resolution transmission electron microscopy (HRTEM) was carried out to further characterize the microstructure of the RuP_2_/Mn_2_P_2_O_7_/NPC. Specifically, the particle size distribution presented in Figure 1e was obtained by analyzing more than 100 nanoparticles from randomly selected regions of high-resolution TEM images using Nano Measurer 1.2.5 software. RuP_2_/Mn_2_P_2_O_7_ nanoparticles with an average size of 5.47 nm were generated in the RuP_2_/Mn_2_P_2_O_7_/NPC, and they were highly uniformly distributed on the nitrogen-doped porous carbon framework (insert graph of Figure 1e). The precision fabrication of highly dispersed RuP_2_/Mn_2_P_2_O_7_ nanoclusters benefited from the coordination confinement of P atoms enabling the isolation of the ruthenium and manganese precursors, effectively confining their migration and agglomeration during pyrolysis. Moreover, Figure 1f distinctly reveals the coexistence of two well-defined phases along with their interfacial region. More specifically, the HRTEM analysis quantitatively confirmed the lattice spacings of 0.396 nm and 0.261 nm in the RuP_2_/Mn_2_P_2_O_7_/NPC composite, which were unambiguously assigned to the (110) plane of RuP_2_ (PDF#71-0167) and the (130) plane of Mn_2_P_2_O_7_ (PDF#77-1244), respectively. This crystallographic evidence not only validates the phase composition but also provides direct visualization of the heterointerface between the RuP_2_ and Mn_2_P_2_O_7_ components. In addition, the SEM image and corresponding energy-dispersive X-ray spectroscopy (EDS) elemental mapping images confirm the homogeneous distribution of Ru, Mn, P, C, N, and O elements throughout the RuP_2_/Mn_2_P_2_O_7_/NPC catalyst (Figure 1g,h). These characterization results collectively demonstrate that the RuP_2_/Mn_2_P_2_O_7_/NPC catalyst exhibits a three-dimensional porous framework and RuP_2_/Mn_2_P_2_O_7_ heterojunction active sites.

### 3.2. Structural Analysis of the Materials

**Figure 2:**
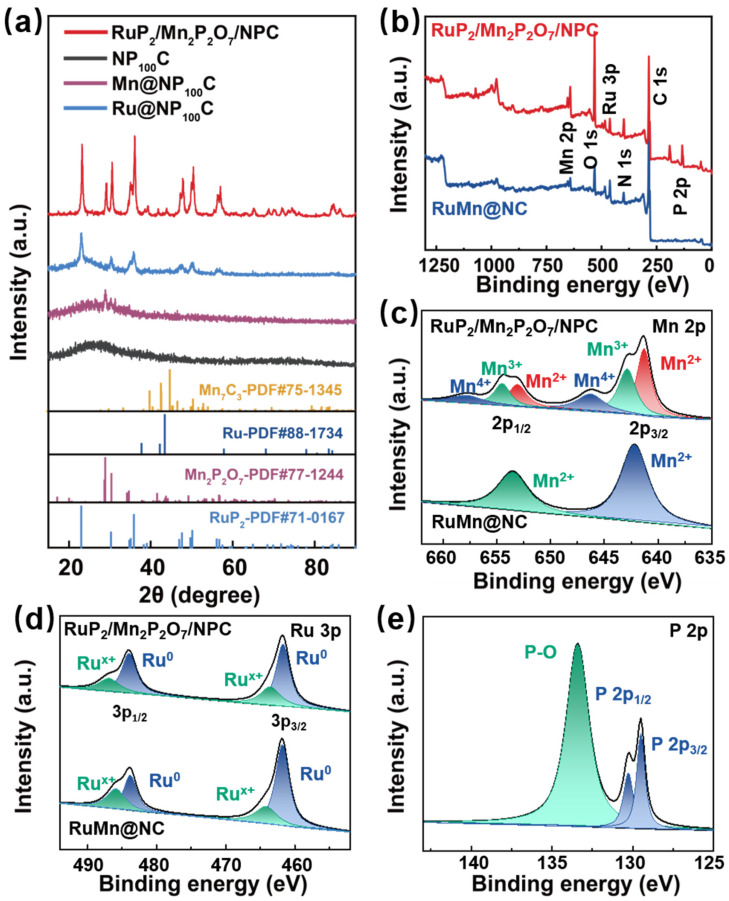
(**a**) PXRD patterns of the obtained catalysts. (**b**) XPS spectra of RuP_2_/Mn_2_P_2_O_7_/NPC and RuMn@NC. (**c**) Mn 2p, (**d**) Ru 3p, and (**e**) P 2p XPS spectra of RuP_2_/Mn_2_P_2_O_7_/NPC.

To investigate the structural information on and lattice parameters of the RuP_2_/Mn_2_P_2_O_7_/NPC catalysts, we conducted PXRD characterizations. As depicted in Figure 2a, the XRD pattern of the RuP_2_/Mn_2_P_2_O_7_/NPC exhibits several sharp peaks at 23.3°, 30.5°, 35.0°, 36.0°, 38.5°, 39.2°, 47.2°, 47.9°, 49.9°, 50.3°, 56.3°, 56.9°, 65.1°, 68.7°, 72.2°, 74.4°, 84.5°, and 86.3°, corresponding to the standard card (PDF#71-0167), demonstrating the successful preparation of RuP_2_ species. Additionally, the five distinct peaks at 0.2°, 29.1°, 41.7°, 43.8°, and 70.7°, which are attributed to the (001), (021), (131), (−311), and (−351) crystal planes of Mn_2_P_2_O_7_ (PDF#77-1244), indicate the existence of Mn_2_P_2_O_7_ species in the RuP_2_/Mn_2_P_2_O_7_/NPC. The absence of prominent Mn_2_P_2_O_7_ characteristic peaks in the PXRD patterns of both the RuMn@NP_150_C and the RuMn@NP_50_C confirms the absence of Mn_2_P_2_O_7_ species in both catalysts (Figure S1). These results demonstrate that the concentration of the phosphorus doping plays a critical role in determining the RuP_2_/Mn_2_P_2_O_7_ heterojunction of the catalyst. For the RuMn@NC sample, the PXRD analysis revealed a series of distinct diffraction peaks at 38.3°, 40.5°, 46.2°, 69.4°, and 86.2° (Figure S1). These reflections exhibit relatively strong intensity and sharp features, suggesting a good degree of crystallinity. While these peaks partially overlap with the characteristic planes of Mn_7_C_3_ (PDF#75-1345), a definitive phase attribution remains tentative due to possible phase mixing and structural complexity. Moreover, additional diffraction peaks appear at 42.2°, 57.8°, 78.0°, 83.5°, and 84.8°, which are close to but slightly right-shifted compared to the (002), (102), (103), (112), and (201) reflections of the metallic Ru (PDF#88-1734). This peak shift may indicate lattice distortion or subtle variations in local crystal fields, possibly arising from Mn incorporation or heteroatomic interactions within the structure. These results suggest that the RuMn@NC sample primarily contains crystalline Ru phases, with possible Mn-C species co-existing in a complex microstructure.

Furthermore, the PXRD patterns of the Mn@NP_100_C and Ru@NP_100_C catalysts reveal different levels of crystallinity and phase purity. For the Mn@NP_100_C, only several weak and broad diffraction peaks were observed, which were tentatively attributed to the Mn_2_P_2_O_7_ species with relatively poor crystallinity, and their positions correspond to the standard Mn_2_P_2_O_7_ pattern (PDF#77-1244). In contrast, the PXRD pattern of the Ru@NP_100_C exhibits sharp and intense peaks that match well with the reference pattern of RuP_2_ (PDF#89-2743), confirming its high phase purity and crystallinity. This contrast further supports the successful construction of the RuP_2_/Mn_2_P_2_O_7_/NPC heterojunction. The presence of Ru during synthesis appears to promote the formation and crystallization of Mn_2_P_2_O_7_, likely due to localized thermal effects from exothermic phosphidation, enhanced lattice compatibility, and a modified redox environment. These synergistic effects contribute to the improved structural ordering and phase integration observed in the heterojunction system [38,39]. The PXRD pattern of the NP_100_C exhibits a broad diffraction feature centered around 23°, corresponding to the typical amorphous carbon structure, while no clear reflections associated with graphitic ordering were observed, suggesting a low degree of carbon framework ordering and the predominance of disordered carbon domains in the NP_100_C sample.

To further investigate electronic interactions at the heterointerface of RuP_2_/Mn_2_P_2_O_7_/NPC, the chemical environment and bonding configurations of C, N, P, Ru, Mn, and O in the RuP_2_/Mn_2_P_2_O_7_/NPC catalysts were examined using XPS. For the comparison, the electronic structure of the RuMn@NC was employed. The full XPS survey spectra of the RuP_2_/Mn_2_P_2_O_7_/NPC and RuMn@NC (Figure 2b) reveal distinct elemental compositions at the surface. The RuP_2_/Mn_2_P_2_O_7_/NPC exhibits six characteristic elements (Mn, Ru, P, C, N, and O), while the RuMn@NC shows five primary elements (Mn, Ru, C, N, and O), with the absence of phosphorus being particularly notable. In the Mn 2p XPS spectra, the peaks of the Mn 2p_3/2_ were observed at 641.3 eV, 642.8 eV, and 646.2 eV, corresponding to Mn^2+^, Mn^3+^, and Mn^4+^ species for the RuP_2_/Mn_2_P_2_O_7_/NPC, indicating a higher oxidation state of Mn species in the RuP_2_/Mn_2_P_2_O_7_/NPC compared to the RuMn@NC (Mn^2+^) (Figure 2c). Additionally, in the RuP_2_/Mn_2_P_2_O_7_/NPC, the Ru 3p XPS analysis also indicates the existence of multiple valence states: the peaks located at binding energies of 461.7 eV and 483.9 eV are attributed to the Ru 3p_3/2_ and 3p_1/2_ orbitals, respectively, whereas the new peaks at 463.5 eV and 486.8 eV represent the presence of the Ru^x+^ (0 < x < 4) species, suggesting that Ru not only exists in the zero-valence state, but also partially oxidizes to form a high-valence state. In contrast, the RuMn@NC shows two different binding energies for Ru species, Ru^0^ (461.8 eV) and Ru^x+^ (464.1 eV), corresponding to the Ru 3p_3/2_ orbitals (Figure 2d). In the Ru 3p XPS spectra, the Ru^x+^ species in the RuP_2_/Mn_2_P_2_O_7_/NPC shows a negative shift of 0.6 eV compared to the RuMn@NC, indicating the remodeling effect of the heterostructure on the electronic configuration. In the P 2p spectrum (Figure 2e), three distinct subpeaks were observed: 130.2 eV and 129.4 eV correspond to the 2p_1/2_ and 2p_3/2_ orbitals of P in the metal phosphide, respectively, whereas the broad peak at 133.4 eV was attributed to the presence of P-O bonding, confirming the formation of the P-O coordination structure and indirectly verifying the effective doping of the P element. These XPS results reveal that constructing a RuP_2_/Mn_2_P_2_O_7_ heterojunction serves as an effective strategy for manipulating the local electronic environment of Ru and Mn centers.

**Figure 3:**
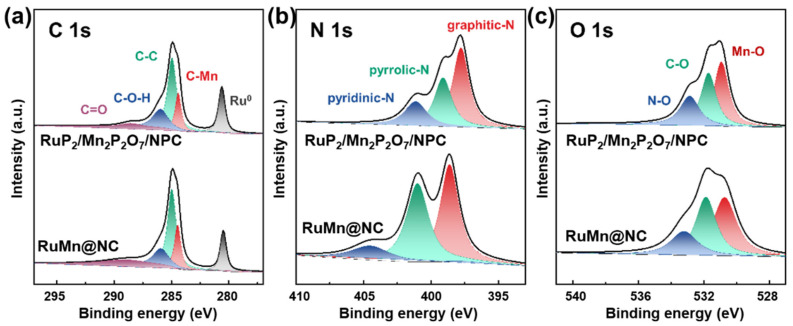
(**a**) C 1s, (**b**) N 1s, and (**c**) O 1s XPS spectra of RuP_2_/Mn_2_P_2_O_7_/NPC and RuMn@NC.

Furthermore, the XPS analysis of the C, N, and O elements confirmed the presence of interfacial electronic interactions between the carbon substrate and the metal components. In the C 1s spectra (Figure 3a) of the RuP_2_/Mn_2_P_2_O_7_/NPC, five distinct peaks were observed at 288.7 eV, 286.0 eV, 284.9 eV, and 284.4 eV, which were assigned to the C=O, C-O-H, C-C, and C-Mn (284.4 eV), and, notably, a peak at 280.6 eV, which was attributed to Ru-C interactions. These findings indicate strong electronic coupling between the metal species (Ru and Mn) and the carbon support. In the N 1s spectrum (Figure 3b), the RuP_2_/Mn_2_P_2_O_7_/NPC exhibited peaks corresponding to pyridinic-N (397.8 eV), pyrrolic-N (399.1 eV), and graphitic-N (401.1 eV). Compared to the RuMn@NC (in which these peaks appeared at 398.6, 401.0, and 404.6 eV, respectively), the binding energy in the RuP_2_/Mn_2_P_2_O_7_/NPC was significantly negatively shifted, which suggests that P-doping increases the electron density around N atoms, thereby enhancing the material’s overall electron transport capability. Nitrogen, primarily introduced from melamine precursors, regulates catalytic performance by modulating the carbon matrix’s electronic structure, thereby improving conductivity and charge transfer. Nitrogen species (e.g., pyridinic, pyrrolic, and graphitic N) also serve as coordination sites, stabilizing metal species and promoting dispersion. Additionally, nitrogen doping increases defect density and strengthens metal–carbon interactions, contributing to structural integrity and long-term durability. Furthermore, the O 1 s spectra (Figure 3c) further support the above conclusion, exhibiting three distinct peaks at 530.9 eV (Mn-O), 531.7 eV (C-O), and 532.9 eV (N-O). The appearance of the Mn-O peak serves as evidence for the formation of a stable Mn–O coordination bond, which enhances the structural integrity of the heterostructure. The elemental composition analysis of RuP_2_/Mn_2_P_2_O_7_/NPC and RuMn@NC is presented in Table 1. Comparative XPS studies demonstrate that phosphorus doping induces substantial modifications in the surface elemental distribution of the RuP_2_/Mn_2_P_2_O_7_/NPC compared to the undoped RuMn@NC. These findings highlight how controlled heterostructure engineering can effectively modulate active sites and electronic structures through coordinated structural and compositional adjustments. In summary, the RuP_2_/Mn_2_P_2_O_7_ heterojunction on carbon substrates demonstrates synergistic stability, with phosphorus doping effectively modulating the electronic configuration and strengthening interfacial interactions. These modifications collectively boost the catalytic performance of RuP_2_/Mn_2_P_2_O_7_/NPC.

### 3.3. Electrochemical Performance of the Catalyst for HER

**Figure 4:**
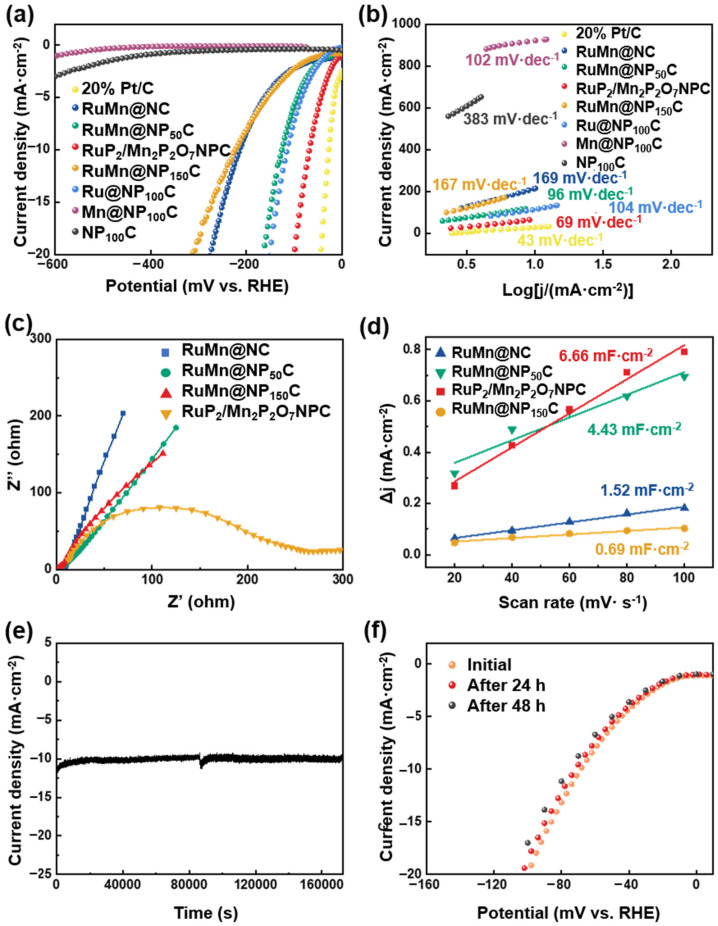
Electrocatalytic performance for HER in alkaline solution. (**a**) LSV polarization curves and (**b**) Tafel slope of RuP_2_/Mn_2_P_2_O_7_/NPC, RuMn@NC, RuMn@NP_50_C, RuMn@NP_150_C, Ru@NP_100_C, Mn@NP_100_C, NP_100_C, and 20% Pt/C. (**c**) EIS Nyquist plots of RuP_2_/Mn_2_P_2_O_7_/NPC, RuMn@NC, RuMn@NP_50_C, and RuMn@NP_150_C. (**d**) C_dl_ curves of RuMn@NC, RuMn@NP_150_C, RuMn@NP_50_C, and RuP_2_/Mn_2_P_2_O_7_/NPC catalysts. (**e**) i-t curve of RuP_2_/Mn_2_P_2_O_7_ catalyst stability test at 69 mV vs. RHE. (**f**) Comparison of polarization curves of RuP_2_/Mn_2_P_2_O_7_/NPC before and after 48 h.

To evaluate the HER catalytic activity of the RuP_2_/Mn_2_P_2_O_7_/NPC, electrochemical experiments were conducted with 1 M KOH, employing a conventional three-electrode system. For comparison, several catalysts were measured, including RuMn@NC, RuMn@NP_150_C, RuMn@NP_50_C, Mn@NP_100_C, Ru@NP_100_C, NP_100_C, and commercial Pt/C (20 wt%). The catalyst ink was prepared by dispersing the synthesized powder in a mixture of water, isopropanol, and a small amount of Nafion binder. This ink was drop-cast onto a polished glassy carbon electrode, forming a uniform and adherent catalyst layer. Nafion serves as an effective adhesive, enhancing mechanical stability, while the porous carbon framework provides physical interlocking with the electrode. Electrochemical tests, including repeated voltammetric scans and chronoamperometry, demonstrated consistent current responses, confirming the catalyst layer’s robustness throughout the measurements. The HER performance of the prepared catalysts was first assessed using polarization curves with 95% iR compensation. As shown in Figure 4a, the RuP_2_/Mn_2_P_2_O_7_/NPC exhibits excellent HER activity, requiring a low overpotential of only 69 mV to reach a current density of 10 mA·cm^−2^, approaching the performance of 20 wt% Pt/C (30 mV), which was significantly lower than that of the single-metal phosphide catalysts, including Mn@NP_100_C, Ru@NP_100_C (115 mV), and NP_100_C, clearly indicating that both metal phosphide phases contributed active sites and that their synergistic interaction greatly enhanced the intrinsic HER performance. In addition, RuP_2_/Mn_2_P_2_O_7_/NPC demonstrates exceptional HER performance in terms of overpotential, higher than those of the previously reported non-noble metal catalysts (Table S1). Notably, RuP_2_/Mn_2_P_2_O_7_/NPC shows superior HER performance to that of non-phosphidated RuMn@NC (215 mV), RuMn@NP_150_C (224 mV), and RuMn@NP_50_C (126 mV), demonstrating that the constructed RuP_2_/Mn_2_P_2_O_7_ heterojunction serves as the dominant factor governing the enhanced HER catalytic activity. Furthermore, the Tafel slope analysis demonstrated significantly enhanced HER kinetics for RuP_2_/Mn_2_P_2_O_7_/NPC (69 mV·dec^−1^), exhibiting a reduction in Tafel slope of approximately 60–80% compared to the RuMn@NC (169 mV·dec^−1^), RuMn@NP_50_C (96 mV·dec^−1^), RuMn@NP_150_C (167 mV·dec^−1^), Ru@NP_100_C (104 mV·dec^−1^), Mn@NP_100_C (102 mV·dec^−1^), and NP_100_C (383 mV·dec^−1^) (Figure 4b). Additionally, an electrochemical impedance spectroscopy (EIS) analysis was carried out to evaluate the kinetic performance of the catalysts. As depicted in Figure 4c, the RuP_2_/Mn_2_P_2_O_7_/NPC exhibits a smaller charge transfer resistance than those of the RuMn@NC, RuMn@NP_50_C, and RuMn@NP_150_C, indicating faster charge transfer in RuP_2_/Mn_2_P_2_O_7_/NPC relative to the RuP_2_/Mn_2_P_2_O_7_ heterojunction. Electrochemical double-layer capacitance (C_dl_) measurements (Figure 4d, Figures S2 and S3 and Table S2) in the non-Faradaic region revealed that RuP_2_/Mn_2_P_2_O_7_/NPC exhibits the highest C_dl_ value (6.66 mF·cm^−2^), representing a 438% increase over RuMn@NC (1.52 mF·cm^−2^), a 150% enhancement compared to RuMn@NP_50_C (4.43 mF·cm^−2^), a 965% improvement relative to RuMn@NP_150_C (0.69 mF·cm^−2^), and superiority to counter catalysts (Ru@NP_100_C: 3.58 mF·cm^−2^, Mn@NP_100_C: 2.32 mF·cm^−2^, and NP_100_C: 1.78: mF·cm^−2^), indicating its superior electrochemically active surface area. Long-term operational durability represents a fundamental requirement for implementing electrocatalytic materials in industrial applications. Hence, the electrochemical durability of the RuP_2_/Mn_2_P_2_O_7_/NPC was systematically evaluated through chronoamperometric measurements at a fixed potential under HER operation conditions. As illustrated in Figure 4e, the current density for the HER remained robust throughout the 48 h stability assessment under a constant potential. In addition, after the 48 h and 100 h i-t stability test, the LSV test on the RuP_2_/Mn_2_P_2_O_7_/NPC catalyst revealed that there was almost no decay at different current densities (Figure 4f and Figure S4). These results demonstrate that the synergistic interplay between RuP_2_ and Mn_2_P_2_O_7_ endows the heterostructure with both catalytically active sites and a robust structure.

## 4. Conclusions

In summary, we have developed a novel RuP_2_/Mn_2_P_2_O_7_ heterojunction integrated into a nitrogen and phosphorus co-doped porous carbon framework (RuP_2_/Mn_2_P_2_O_7_/NPC) as an efficient and durable electrocatalyst for the hydrogen evolution reaction (HER) in alkaline media. The rational design of this heterojunction is achieved through a controlled pyrolysis–phosphidation strategy, which promotes intimate interfacial contact and electronic coupling between RuP_2_ and Mn_2_P_2_O_7_ phases. This synergy effectively modulates the local electronic environment and accelerates charge transfer. As a result, RuP_2_/Mn_2_P_2_O_7_/NPC exhibits an overpotential of just 69 mV in order to reach 10 mA·cm^−2^, along with a Tafel slope as low as 69 mV·dec^−1^. Moreover, the catalyst demonstrates superior electrochemical stability, retaining its performance over 48 h of continuous operation. This work underscores the promise of transition metal phosphide heterojunctions for next-generation alkaline HER electrocatalysts and provides a generalizable strategy for interfacial engineering to enhance catalytic functionality in energy conversion applications.

## Figures and Tables

**Table 1:** Elemental composition of RuP_2_/Mn_2_P_2_O_7_/NPC and RuMn@NC.

Element	RuP_2_/Mn_2_P_2_O_7_/NPC (Atomic%)	RuMn@NC (Atomic%)
Ru	1.91	2.19
Mn	0.79	3.31
P	11.8	0
N	9.71	5.66

## Data Availability

The original contributions presented in this study are included in the article/Supplementary Material. Further inquiries can be directed to the corresponding author.

## Supplementary Materials

The following supporting information can be downloaded at: https://www.mdpi.com/article/10.3390/ma18133065/s1. Figure S1. PXRD patterns of the counter catalysts. Figure S2. CV curves of (a) RuMn@NC, (b) RuMn@NP_50_C, (c) RuP_2_/Mn_2_P_2_O_7_/NPC, and (d) RuMn@NP_150_C catalysts. Figure S3. CV curves of (a) Ru@ NP_100_C, (b) Mn@NP_100_C, (c) NP_100_C, and (d) C_dl_ curves of Ru@ NP_100_C, Mn@NP_100_C, and NP_100_C catalysts. Figure S4. Stability test of RuP_2_/Mn_2_P_2_O_7_ catalyst at overpotential of 69 mV vs. RHE. Table S1. Performance comparison between RuP_2_/Mn_2_P_2_O_7_/NPC and recently reported catalysts. Table S2. C_dl_ comparison between RuP_2_/Mn_2_P_2_O_7_/NPC and counter catalysts.
