# Supplementary material for: Interface-Engineered RuP2/Mn2P2O7 Heterojunction on N/P Co-Doped Carbon for High-Performance Alkaline Hydrogen Evolution
Source: Materials (Basel). 2025 Jun 27;18(13):3065. doi: 10.3390/ma18133065 (PMC12251403; doi:10.3390/ma18133065)
Supplement: Supplementary file 1 [file materials-18-03065-s001.zip › materials-3694759-supplementary.pdf]

Supporting information

Interface-engineered $RuP_2/Mn_2P_2O_7$ Heterojunction on N/P Co-doped Carbon for High-performance Alkaline Hydrogen Evolution

Wenjie Wu[1], Wenxuan Guo[1], Zeyang Liu[1], Chenxi Zhang[1], Aobing Li[1], Caihua Su[2], Chunxia Wang[1*], and Guoyong Huang[1*]

[1]College of New Energy and Materials, State Key Laboratory of Heavy Oil Processing China University of Petroleum (Beijing), Beijing 102249, China

[2] Beijing Future Hydrogen Technology Co., Ltd, Beijing 102209, China

[*]Authors to whom correspondence should be addressed.

Figures

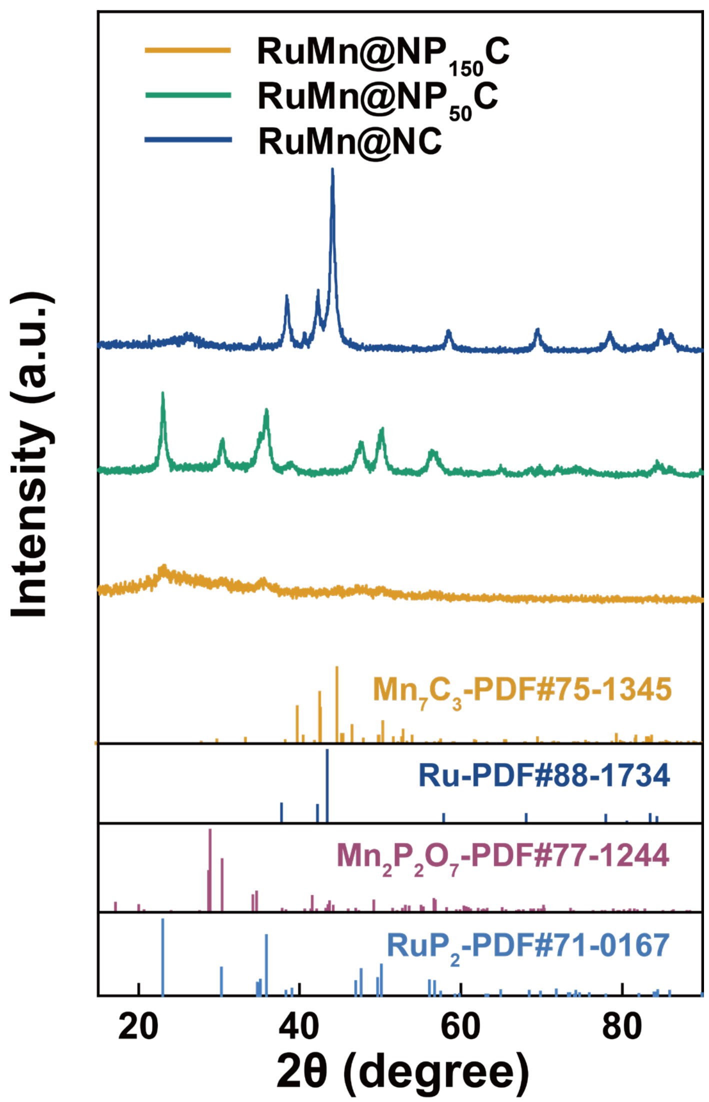

Figure S1. PXRD patterns of the counter catalysts.

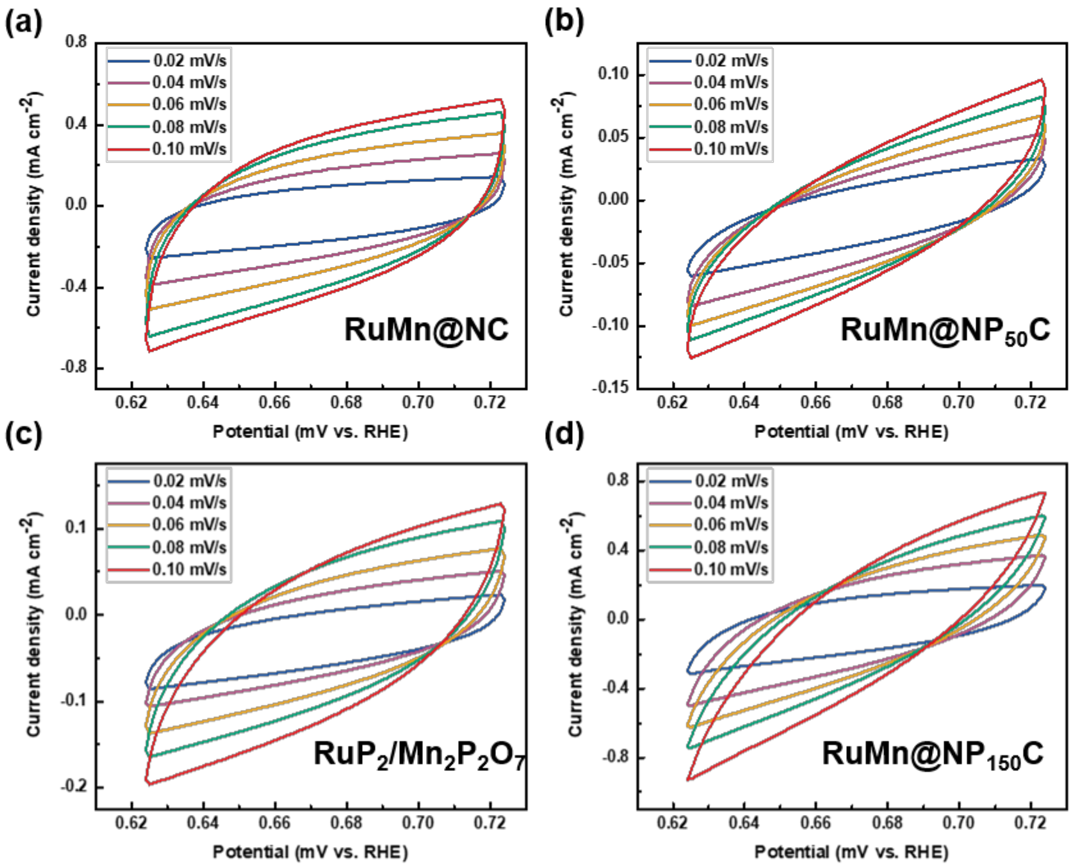

Figure S2. CV curves of (a) RuMn@NC, (b) RuMn@NP$_{50}$C, (c) $RuP_2/Mn_2P_2O_7$/NPC, and (d) RuMn@NP$_{150}$C catalysts

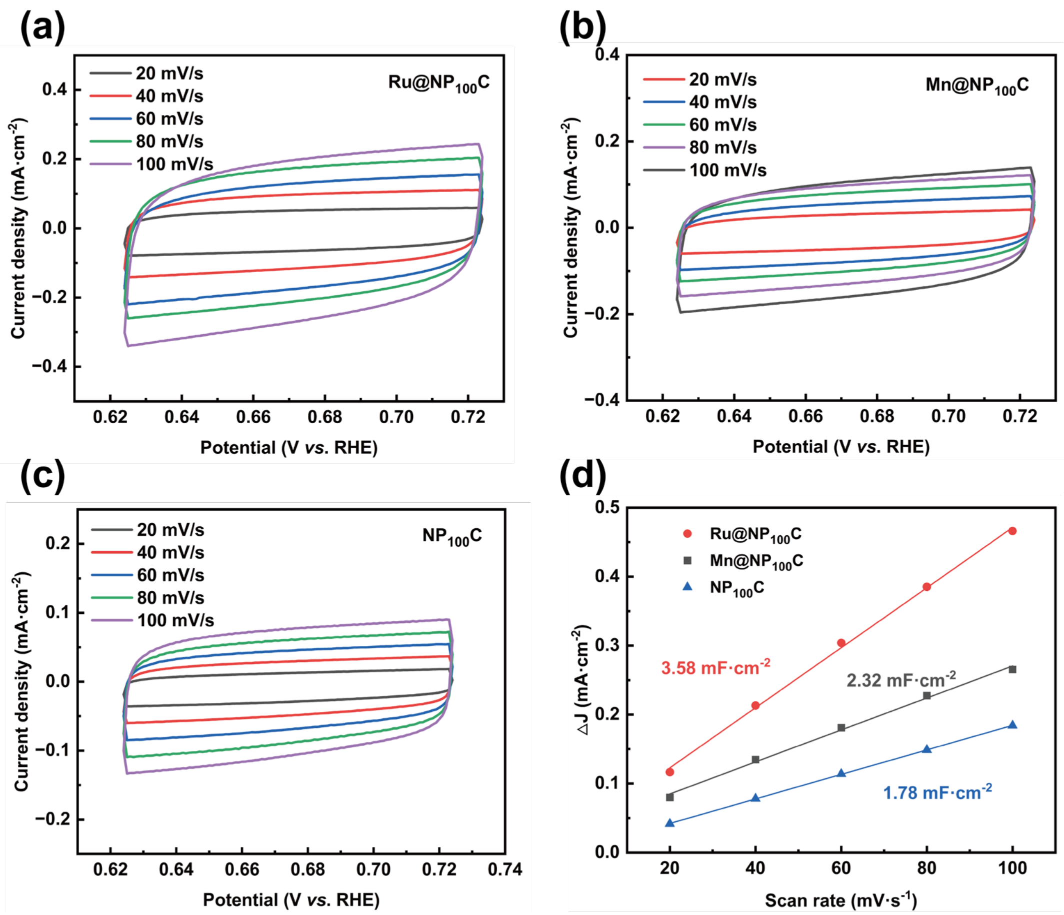

Figure S3. CV curves of (a) Ru@ $NP_{100}C$, (b) Mn@$NP_{100}C$, (c) $NP_{100}C$, and (d) C_{dl} curves of Ru@ $NP_{100}C$, Mn@$NP_{100}C$, and $NP_{100}C$ catalysts.

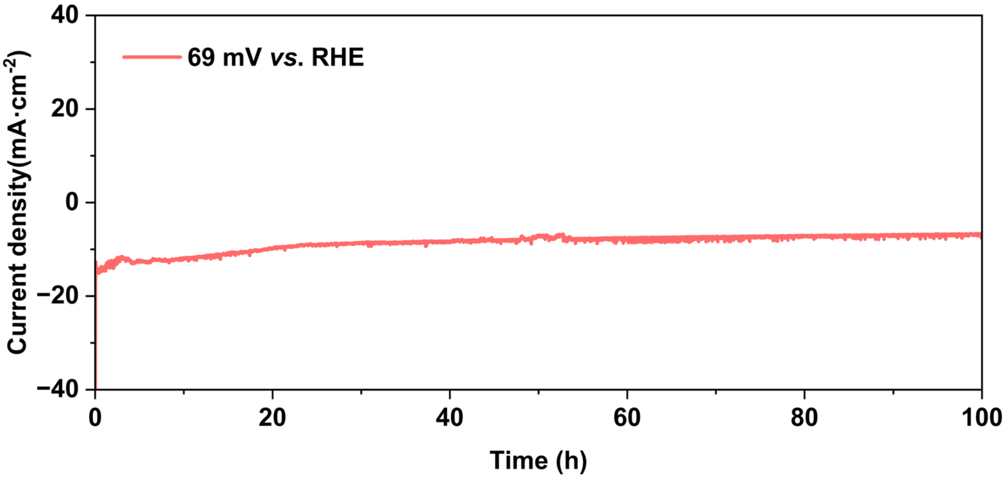

Figure S4. Stability test of $RuP_2/Mn_2P_2O_7$ catalyst at overpotential of 69 mV *vs*. RHE.

Table S1. Performance comparison between $RuP_2/Mn_2P_2O_7$/NPC and recently reported catalysts.

Catalyst	Over potential@10 $mA \cdot cm^{-2}$ (mV *vs*. RHE)	Reference
$RuP_2/Mn_2P_2O_7$/NPC	69	This work
FeP@NPC	109	Appl. Surf. Sci.,2022,597,153662
FeP@PPy/CTs	103.1	Chem. Eng. J., 2022, 433, 133643
MoO_2-FeP@C	103	Adv. Mater., 2020, 32, 2000455
FexNiy/CeO_2/NC	240	Inorg. Chem. Front., 2020, 7, 470
Ce-doped NiFe LDH	147	Sustainable Energy Fuels, 2020, 4, 312
NF@NiFe LDH/CeO_x	154	ACS Appl. Mater. Interfaces, 2018, 10, 35145

Table S2. C_{dl} comparison between $RuP_2/Mn_2P_2O_7$/NPC and counter catalysts.

Catalyst	C_{dl} ($mF \cdot cm^{-1}$)
$RuP_2/Mn_2P_2O_7$/NPC	6.66
RuMn@NC	1.52
RuMn@$NP_{50}C$	4.43
RuMn@$NP_{150}C$	0.69
Ru@ $NP_{100}C$	3.58
Mn@$NP_{100}C$	2.72
$NP_{100}C$	1.78
